# Editorial: Approximate Number System and Mathematics

**DOI:** 10.3389/fpsyg.2019.02084

**Published:** 2019-09-12

**Authors:** Jingguang Li, Xinlin Zhou, Marcus Lindskog

**Affiliations:** ^1^College of Education, Dali University, Dali, China; ^2^State Key Laboratory of Cognitive Neuroscience and Learning, IDG/McGovern Institute for Brain Research, Beijing Normal University, Beijing, China; ^3^Department of Psychology, Uppsala University, Uppsala, Sweden

**Keywords:** approximate number system, number sense, non-symbolic number acuity, numerical cognition, mathematics

Humans process quantity information without the aid of language or symbols to guide a variety of everyday life decisions. The cognitive system that supports this intuitive skill is often referred to as the approximate number system (ANS). It has been argued that the ANS serves as the foundation of the formal symbolic number system—mathematics (Dehaene, 1997). Abundant empirical evidence is supportive of this view: acuity of the ANS is positively correlated with symbolic math performance (Chen and Li, 2014), training of the ANS may cause improvements in symbolic math performance (Bugden et al., 2016), and the ANS and symbolic number processing may share a common neural underpinning (Piazza et al., 2004). However, recently several theories and empirical data cast doubt on the role of the ANS in symbolic math processing (Reynvoet and Sasanguie, 2016; Leibovich et al., 2017). This Research Topic aims to advance our understanding of the underlying mechanisms of the overlap between the ANS and mathematics.

The first portion of this Research Topic centers on the measurement issue of the ANS. Liu et al. demonstrated that regularity of visual features in the non-symbolic numerical task influenced processing of numerical information. For regular patterns of dot arrays, numerosity processing is inhibited; but for random patterns, numerosity information could be extracted independently of visual features. Thus, to measure ANS acuity, it is necessary to avoid regular dot patterns in the non-symbolic numerical task. van Hoogmoed and Kroesbergen suggested that convex hull, the smallest convex polygon that contains an array of dots, could be a plausible confounding factor in the non-symbolic numerical task. By using event-related potentials (ERP) from electroencephalography recordings, they found no signs of a distance effect for numerosity, but a distance effect for convex hull instead. Consequently, non-numerical visual features might at least partly influence performance in non-symbolic numerical tasks. Hence, it is unclear whether non-numerical visual processing or numerical processing in the non-symbolic numerical task contributes to the widely reported association between ANS acuity and math performance. Furthermore, their ERP data indicated that symbolic and non-symbolic numerosties where processed differentially, questioning if non-symbolic and symbolic numerosities share the same neural circuitry, as previously suggested (e.g., Dehaene, 1997). Braham et al. addressed this issue by using hierarchical linear modeling, which has the advantage of being able to isolate the numerical and non-numerical visual component in non-symbolic numerical task performance both within and between individuals. Critically, they found that only the numerical component contributed to adults' math ability. Finally, Guillaume and Van Rinsveld performed a meta-analysis regarding the variability of the Weber fraction in different versions of the non-symbolic number comparison paradigm. They found that different methods used for controlling for non-numerical information cause highly variable Weber fraction scores. Accordingly, they recommended not to compare Weber fraction scores from different tasks.

The second portion of this Research Topic focuses on the correlation between ANS acuity and math ability. Testing this correlation is the first step for further investigation of the causal relationship between ANS and math performance. Starr et al. suggested a new path underlying the association between ANS and math performance. They found that ANS manipulability (i.e., the ability to perform arithmetic operations on approximate numerical quantities) positively predicted math achievement in preschool children, and the predictive power of ANS manipulability was independent of the influence of ANS acuity. Wei et al. examined the relationship between number magnitude processing and symbolic approximate arithmetic performance (i.e., the ability to provide an approximate answer to an arithmetic question), which should arguably be largely uninfluenced by language. They found that both semantic and spatial number processing (indexed by the two-digit number comparison and number-line estimation task, respectively) are positively correlated to the symbolic approximate arithmetic performance, and these associations are moderated by the task difficulty of the symbolic approximate arithmetic task.

Two studies demonstrated that the correlation between ANS acuity and math performance is moderated by multiple factors. Cai et al. found that the correlation between ANS acuity and math performance varies across different grade levels (kindergarten vs. primary school), type of math tests, and type of ANS tests (non-symbolic estimation vs. number-line task). Using latent class modeling, Chew et al. identified four different magnitude ability profiles based on children's performance in the non-symbolic and symbolic numerical task. Further, they observed both stability and change in the four different profiles across a 1-year time period. Finally, profile membership was differentially related math performance at different ages.

Another two studies revealed that differences in math ability of different populations could be attributed to differences in ANS acuity. Lonnemann et al. found that Chinese children have better counting skills than their German peers. More importantly, the advantages in counting in Chinese children were accompanied by superior performance in a non-symbolic numerical comparison task. In addition, Oliveira et al. reported a case study on a girl with specific numerical processing impairment and a rare genetic disorder−22q11.2 deletion syndrome. The girl has normal general intelligence; however, she manifested severe deficits in single-digit calculation accompanied by poor performance in the non-symbolic numerical comparison task.

The third portion of this Research Topic examines whether training of the ANS leads to improvement in symbolic math performance. The training approach not only tests the causal relationship between ANS acuity and math performance, but also provides valuable insights for math education (Bugden et al., 2016). Szkudlarek and Brannon found a transfer effect from ANS training to math performance. A group of preschool children trained for 1 month with a computer-based non-symbolic arithmetic training program. After controlling for confounding factors, children with low math abilities in the ANS-training group outperformed control-group children on informal symbolic math problems. In contrast, Kim et al. did not find a transfer effect in their training experiment with first-grade children. Although significant improvement in ANS acuity was observed following a 6-week training period, children showed no improvement in math performance. To resolve the discrepancies between the above two training studies, more replication studies with rigorous methodologies are needed (Szucs and Myers, 2017).

The final portion of this Research Topic examines the distinction and mapping between the ANS and the symbolic numerical processing system by analyzing psychophysical features of different non-symbolic and symbolic numerical tasks. Krajcsi et al. made an extensive comparison of the several psychophysical properties of non-symbolic and symbolic number comparison, including error rates, reaction times, and diffusion-model drift rates. They found that the ratio-based ANS model only fits the non-symbolic number comparison data, but not the symbolic comparison data. Accordingly, the authors argued that different cognitive systems are in charge of symbolic and non-symbolic number processing. Chesney and Matthews found that different versions of non-symbolic numerosity tasks give rise to differences in performance. More specifically, while a free estimation task showed a classical pattern of scalar variability there was no evidence for this error pattern in a number-line and ratio estimation task. Furthermore, participants showed underestimation in the free estimation task but accurate estimation in the ratio task. They argued that these task constraints affect the ANS-math mapping process.

Taken together, this Research Topic combines diverse methodologies to advance our understanding of the relationship between the approximate number system and mathematics. According to the new data in this Research Topic, it might be too simple to conclude that the ANS and math are related or separated. Instead, it is worth asking how (i.e., the cognitive paths) and when (i.e., different developmental stages, task variants, and types of participants) the ANS is linked to math.

## Author Contributions

JL wrote the first draft. JL, XZ, and ML contributed to the revision of the paper.

### Conflict of Interest Statement

The authors declare that the research was conducted in the absence of any commercial or financial relationships that could be construed as a potential conflict of interest.
